# Engineering CO_2_-Fixing Carboxysome into *Saccharomyces cerevisiae* to Improve Ethanol Production

**DOI:** 10.3390/ijms26199759

**Published:** 2025-10-07

**Authors:** Mengqi Li, Simin Zeng, Yunling Guo, Jie Ji, Qiuling Fan, Deqiang Duanmu

**Affiliations:** 1 National Key Laboratory of Agricultural Microbiology, Hubei Hongshan Laboratory, Huazhong Agricultural University, Wuhan 430070, China; 2020304010007@webmail.hzau.edu.cn (M.L.); sm.zeng@siat.ac.cn (S.Z.); yunlingguo@webmail.hzau.edu.cn (Y.G.); jieji@webmail.hzau.edu.cn (J.J.); 2 College of Life Science and Technology, Huazhong Agricultural University, Wuhan 430070, China; qlfan@mail.hzau.edu.cn; 3 Shenzhen Branch, Guangdong Laboratory for Lingnan Modern Agriculture, Genome Analysis Laboratory of the Ministry of Agriculture, Agricultural Genomics Institute at Shenzhen, Chinese Academy of Agricultural Sciences, Shenzhen 518108, China

**Keywords:** bacterial microcompartment, α-carboxysome, carbon dioxide fixation, bioethanol production, *Halothiobacillus neapolitanus*, *Saccharomyces cerevisiae*

## Abstract

Bacterial microcompartments (BMCs) are intracellular structures for compartmentalizing specific metabolic pathways in bacteria. As a unique type of BMCs, carboxysomes utilize protein shells to sequester ribulose-1,5-bisphosphate carboxylase/oxygenase (Rubisco) and carbonic anhydrase for efficient carbon dioxide (CO_2_) fixation. This study aims to reconstruct an α-carboxysome in *Saccharomyces cerevisiae* and investigate its metabolic effects. Here, genes of the *cso* operon from *Halothiobacillus neapolitanus*, Calvin cycle-related enzyme phosphoribulokinase (PRK) from *Spinacia oleracea*, and two *S. cerevisiae* chaperone genes, *HSP60* and *HSP10*, were introduced into *S. cerevisiae*. The engineered yeast strain demonstrated assembled and enzymatically active Rubisco, significant increase in ethanol production and reduction in the byproduct glycerol. Formation of the α-carboxysome structures was observed after purification by sucrose density gradient centrifugation. The engineered yeast strain harboring functional α-carboxysome has the potential for enhancing bioethanol production.

## 1. Introduction

Subcellular organelles of eukaryotic cells enable spatial and temporal control of a variety of biochemical reactions and provide effective means to minimize the inhibitory effect of toxic intermediates and increase the efficiency of compartmentalized metabolic pathways [1,2]. Compartmentalization also occurs in prokaryotic cells. A particular example are the bacterial microcompartments (BMCs) that are composed entirely of proteins, including proteinaceous shells and encapsulated internal enzymes that catalyze sequential reactions. Notably, BMCs were found to be present in twenty-three different bacterial phyla [3].

Carboxysomes are a unique type of BMCs, consisting of α- and β-carboxysomes. The α-carboxysomes exist in α-cyanobacteria and some chemoautotrophs and are specialized in facilitating CO_2_ fixation [4]. The proteinaceous shell of α-carboxysome consists of thousands of protein subunits that encapsulate the CO_2_-fixing enzyme ribulose-1,5-bisphosphate carboxylase/oxygenase (Rubisco) and carbonic anhydrase (CA). The compartmentalization of these enzymes, along with the selective permeability of the shell that facilitates the diffusion of HCO_3_^−^ while reducing CO_2_ leakage, creates a localized high concentration of CO_2_ near Rubisco. This spatial arrangement promotes elevated carboxylation rates of Rubisco within carboxysomes, leading to enhanced carbon fixation [4]. Typically, the α-carboxysome consists of 8–11 different polypeptides. The α-carboxysome shells are mainly composed of CsoS1 hexamers forming the shell faces and CsoS4 pentamers occupying the apex of the polyhedral shells, and both hexamers and pentamers contain multiple homologous proteins [5,6]. The highly abundant intrinsically disordered protein CsoS2 acts as a linker, binding to Rubisco and the shell through its N-terminal and C-terminal domains, respectively [7]. CsoS2 is essential for the α-carboxysome biogenesis and the assembly of the complete α-carboxysome shell [8]. It has been hypothesized that α-carboxysome shells assemble simultaneously or independently as Rubisco proteins aggregate [6,9].

The α-carboxysome of the chemoautotrophic bacterium *H. neapolitanus* has been intensively studied for the purpose of expression in heterologous hosts. The α-carboxysome-like structure was reconstituted in *Escherichia coli* and *Corynebacterium glutamicum* by expressing the *cso* operon of *H. neapolitanus* [10,11,12]. Carboxysome shells provide an oxygen-limiting internal environment that is optimal for oxygen sensitive enzymes, i.e., the H_2_-production capacity of hydrogenase was significantly enhanced in *E. coli* by coexpression of α-carboxysome proteins (CsoS2, CsoS4AB, CsoS1CAB, CsoS1D) [8]. Furthermore, a minimal α-carboxysome shell composed of only CsoS1A and CsoS4A of *H. neapolitanus* was able to be formed in *E. coli* [13].

Many metabolic engineering strategies to develop CO_2_-fixing *S. cerevisiae* strains have been reported [14]. One approach is to heterologously express the Calvin–Benson–Bassham (CBB) cycle-related enzymes in yeast. The expression of Calvin cycle enzymes, specifically phosphoribulokinase (PRK) and Rubisco, in *S. cerevisiae* allows for the reaction of CO_2_, which is a significant byproduct of alcoholic fermentation, with Ribulose 1,5-bisphosphate (RuBP) to produce 3-phosphoglycerate. This compound could be converted to phosphoenolpyruvate (PEP) and ultimately lead to ethanol production. Heterologous expression of form-II Rubisco (L_8_) and PRK in *S. cerevisiae* improved ethanol production [15]. A xylose-fermenting *S. cerevisiae* was engineered to achieve biofuel production by expressing form-II Rubisco (L_8_ or L_2_) or form-I Rubisco (L_8_S_8_)-PRK module [16,17]. However, there are no studies reporting on the generation of α-carboxysome structures to provide the low oxygen environment for CO_2_ fixation in *S. cerevisiae*.

Ethanol is a versatile and fundamental chemical with applications across various industries. *S. cerevisiae* is a preferred producer due to its high ethanol yield and robustness. However, its dependence on sugar and associated metabolic traits have detrimental effects on fermentation performance. To overcome the inherent limitations of sugar dependency, this study developed engineered *S. cerevisiae* strains capable of carbon dioxide fixation by expressing several α-carboxysome-related genes from *H. neapolitanus* and a CBB cycle-related gene encoding PRK from *Spinacia oleracea*. The engineered strain showed apparent Rubisco activity and significant increase in ethanol production. Moreover, the resulting strain demonstrated the presence of α-carboxysome-like structures in the cell. Reconstruction of a functional CO_2_-fixing α-carboxysome in yeast holds potential for synthesizing valuable bioproducts in a sustainable way.

## 2. Results

### 2.1. Heterologous Expression of α-Carboxysome Genes in S. cerevisiae

**Figure 1:**
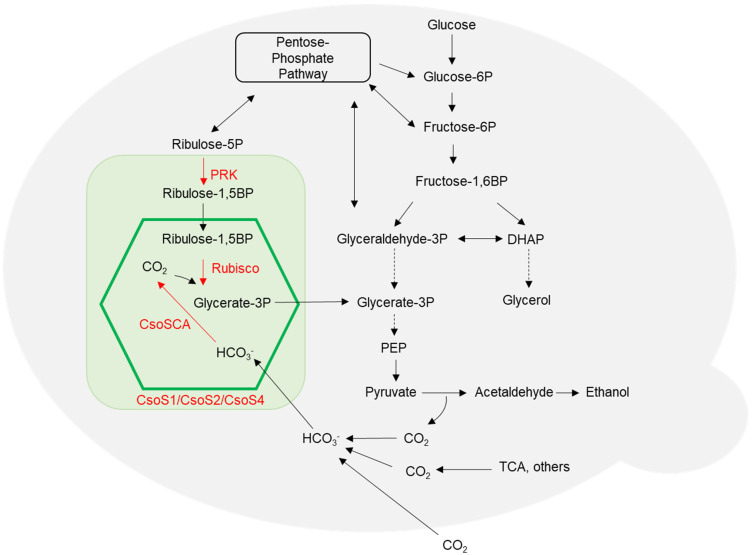
Engineered *S. cerevisiae* metabolic pathways via heterologous expression of α-carboxysome genes and genes encoding metabolic enzymes. The green box represents the heterologous pathway, including carboxysome proteins (CsoS1, CsoS2 and CsoS4) and metabolic enzymes PRK (*S. oleracea* phosphoribulokinase), Rubisco (*H. neapolitanus* Ribulose-1,5-bisphosphate carboxylase/oxygenase) and CsoSCA (*H. neapolitanus* carbonic anhydrase). The carboxysome is illustrated by a green hexagon. The carboxysome shell is permeable to Ribulose-1,5BP, Glycerate-3P and HCO_3_^−^. Abbreviations: DHAP, dihydroxyacetone phosphate; PEP, phosphoenolpyruvate; TCA, tricarboxylic acid cycle. Solid arrows represent direct reactions. Dotted arrows represent multiple reactions.

**Figure 2:**
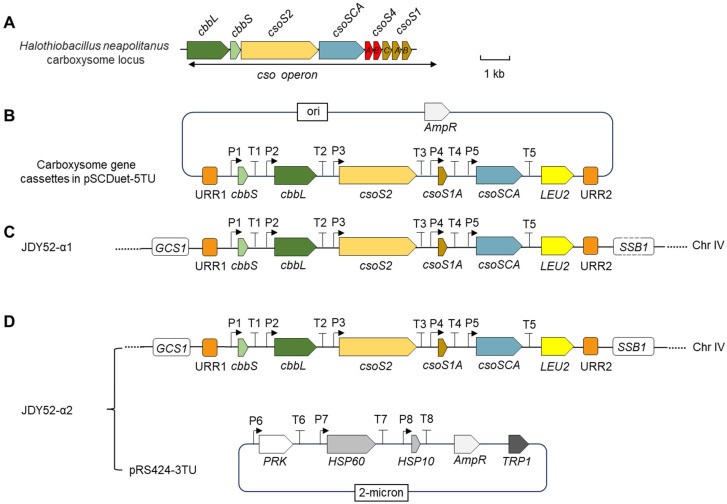
Illustration of heterologous expression of α-carboxysome related genes in *Saccharomyces cerevisiae*. (**A**) The genomic organization of carboxysome locus in *Halothiobacillus neapolitanus. cbbL*, Rubisco large subunit; *cbbS*, Rubisco small subunit; *csoSCA*, carbonic anhydrase gene; *csoS1*, *csoS2* and *csoS4*, carboxysome protein genes. Scale bar, 1 kb. (**B**) Carboxysome gene cassettes in the vector pSCDuet-5TU. Codons of each individual gene (*cbbS*, *cbbL*, *csoS2*, *csoS1A*, *csoSCA*) were optimized for expression in *S. cerevisiae,* and each gene was regulated by specific promoters and terminators. The vector contains a selective marker gene *LEU2* and unique recombination regions (URR1 and URR2). P1, PYK1 promoter; T1, NAT5 terminator; P2, TPI1 promoter; T2, CPS1 terminator; P3, RPL3 promoter; T3, ADH1 terminator; P4, ENO2 promoter; T4, RPL15A terminator; P5, RPL8B promoter; T5, VMA2 terminator; LEU2, leucine selectable marker; URR, homologous recombination arms. (**C**) Genomic organization of the engineered yeast strain JDY52-α1 containing carboxysome-related genes. The cassettes in (**B**) were integrated into the HO locus of yeast strain JDY52-URR by homologous recombination. *GCS1*, growth cold sensitive; *SSB1*, stress-seventy subfamily B. (**D**) Genomic organization of JDY52-α2 by expressing additional three genes in JDY52-α1. The three genes, PRK (phosphoribulokinase of *S. oleracea*, Gene ID: LOC110791203), yeast endogenous HSP60 (heat shock protein 60, SGD: S000004249) and HSP10 (heat shock protein 10, SGD:S000005546) were cloned in the vector pRS424-3TU which was used to transform JDY52-α1. Codons of *PRK* gene were optimized for expression in *S. cerevisiae.* P6, TEF1 promoter; T6, CYC1 terminator; P7, PFK1 promoter; T7, PRC1 terminator; P8, PGK1 promoter; T8, NAT1 terminator. TRP1, tryptophan selectable marker.

*S. cerevisiae* utilizes glycolysis and pentose phosphate pathway (PPP) to metabolize sugar. To develop a *S. cerevisiae* strain which is able to assimilate CO_2_ in a low-oxygen environment, the metabolic pathway was manipulated by introducing *H. neapolitanus* α-carboxysome encapsulating Rubisco and carbonic anhydrase (CsoSCA), the CBB recycle-related enzyme PRK of *Spinacia oleracea*, and endogenous chaperones Hsp60 and Hsp10 into *S. cerevisiae* genome (Figure 1). The strategy is as follows. First, *H. neapolitanus* genes encoding CsoS1A, CsoS2, Rubisco and CsoSCA were expressed in *S. cerevisiae* strain JDY52-URR to develop the strain JDY52-α1 (Figure 2). Next, *S. oleracea PRK* gene and the endogenous chaperone genes (*HSP60*, *HSP10*) were transformed into JDY52-α1 to generate JDY52-α2 (Figure 2).

**Figure 3:**
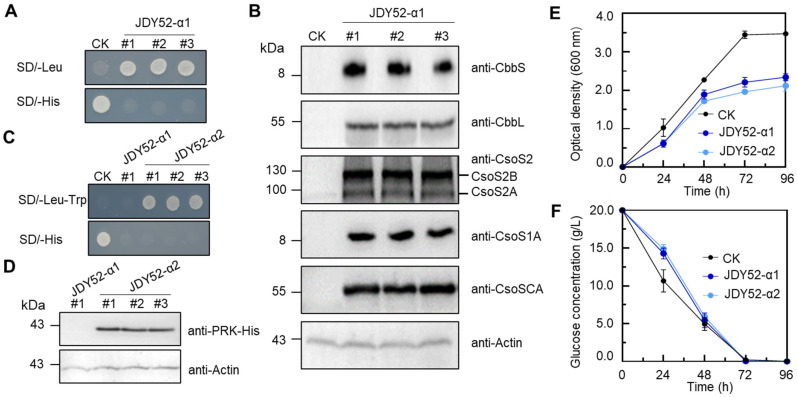
Characterization of engineered *S. cerevisiae* strains JDY52-α1 and JDY52-α2. (**A**,**C**) Growth phenotype of JDY52-α1 and JDY52-α2 on indicated media plates. CK, JDY52-URR as the control. (**B**) Immunoblot analysis of carboxysome genes of three JDY52-α1 strains that were randomly chosen and cultured on SD lacking Leu. The antisera for each protein (CbbS, CbbL, CsoS2, CsoS1A, and CsoSCA) were used to detect the protein abundance. The antisera against CsoS2 were able to detect both CsoS2A (truncated CsoS2) and CsoS2B (full-length CsoS2). Actin was used for equal loading control. (**D**) Immunoblot analysis of PRK protein of yeast strain JDY52-α2. His-tagged PRK protein was detected by anti-His antibody. Actin was used for equal loading control. (**E**) Time–course growth curve of different *S. cerevisiae* strains grown on SC media. Optical density at 600 nm was measured. (**F**) Time–course glucose consumption of *S. cerevisiae* strains grown on SC media. In (**E**,**F**), data represented three biological replicates and error bars indicated standard deviations.

*H. neapolitanus* genes encoding α-carboxysome are mainly clustered in a single *cso* operon, including *cbbL*/*cbbS* encoding Rubisco large and small subunits, *csoSCA*, and shell-encoding genes *csoS2*, *csoS4A*/*B* and *csoS1A*/*B*/*C*/*D*/*E* [6]. The α-carboxysome genetic loci of *H. neapolitanus* are shown in Figure 2A. The α-carboxysome structures encapsulating Rubisco were formed in tobacco chloroplasts after introducing the minimal gene set including two carboxysome genes (*csoS1A* and *csoS2*) and Rubisco genes (*cbbL* and *cbbS*) [18]. In this study, the minimal gene set for the formation of α-carboxysome, together with the *csoSCA* gene, was chosen to assess the feasibility of assembling carboxysome-like structures in *S. cerevisiae*. The carboxysome gene cassette was ligated into a modified vector pSCDuet (Figure 2B) and then integrated into the genome of JDY52-URR [19], generating yeast strain JDY52-α1 (Figure 2C). The JDY52-α1 positive clones were selected on SD medium plates lacking Leu (Figure 3A) and further verified by PCR (Figure S1A,C). The expression of shell protein CsoS1A, scaffolding protein CsoS2, Rubisco large subunit (CbbL) and small subunit (CbbS), and CsoSCA was validated by immunoblot analysis using specific antibodies (Figure 3B). The single carboxysome *csoS2* gene was reported to be able to produce two distinct protein forms due to the -1 ribosomal frameshifting [20]. In Figure 3B, both the full-length CsoS2B and the truncated CsoS2A were detectable, indicating that heterologous expression of *csoS2* gene could also produce two products in *S. cerevisiae*.

### 2.2. Construction of CO_2_-Fixing S. cerevisiae by Expression of Chaperones and Phosphoribulokinase

The yeast mitochondrial chaperonin system consists of HSP60 and HSP10, which play crucial roles in protein folding, sorting, and assembly [21]. HSP60 and HSP10 contain mitochondrial target sequences and are highly conserved across prokaryotes and eukaryotes [22,23]. Purified mitochondrial HSP60 and HSP10 from yeast could mediate the refolding of denatured Rubisco in vitro [24]. Yeast HSP60 and HSP10 proteins or *E. coli* counterpart chaperonin systems GroEL and GroES had been shown to be able to assist Rubisco folding in *S. cerevisiae* and *Pichia pastoris* [14,17,25]. Yeast proteins HSP60 and HSP10 with mitochondrial signal sequences removed were used in this study to ensure the cytosolic localization of HSP60 and HSP10 for the normal folding of Rubisco in *S. cerevisiae*. PRK is one of the key enzymes besides Rubisco to develop a CO_2_-fixing yeast. *S. oleracea* PRK protein was previously successfully expressed in *S. cerevisiae* and *P. pastoris* [17,26,27]. In this study, *S. oleracea PRK* gene was introduced into JDY52-α1 to produce ribulose-1,5-bisphosphate (RuBP).

To generate a functional CO_2_-fixation system in *S. cerevisiae*, a module of *PRK*-*HSP60*-*HSP10* was constructed and introduced into JDY52-α1 to make JDY52-α2 (Figure 2D). PRK and chaperone genes were introduced as a form of plasmid rather than a chromosomally integrated form because of the advantages of easier plasmid construction and selection of transgenic yeast strains. The positive JDY52-α2 clones were selected on SD medium lacking Leu and Trp (Figure 3C) and verified by PCR with different primer pairs (Table S1) to amplify different fragments spanning the module *PRK*-*HSP60*-*HSP10* (Figure S1B,D). PRK protein with His-tag was detectable by immunoblot analysis in JDY52-α2 strains and *S. cerevisiae.* Actin was used as a loading control (Figure 3D). We next characterized JDY52-α1 and JDY52-α2 in terms of cell growth curve and glucose consumption rates in comparison to the control JDY52-URR. In the logarithmic growth phase (24 h and 48 h), the growth of JDY52-α1 and JDY52-α2 was comparable to the control. At the stationary phase (72 h and 96 h) the two engineered strains appeared to reach a lower cellular density than the control (Figure 3E). Those results suggest that expression of multiple genes had an inhibitory effect on yeast growth. As for glucose consumption, the control JDY52-URR showed faster glucose consumption than two engineered strains, but all strains almost depleted glucose after 72 h (Figure 3F).

### 2.3. Functional Expression and Assembly of Rubisco in S. cerevisiae

**Figure 4:**
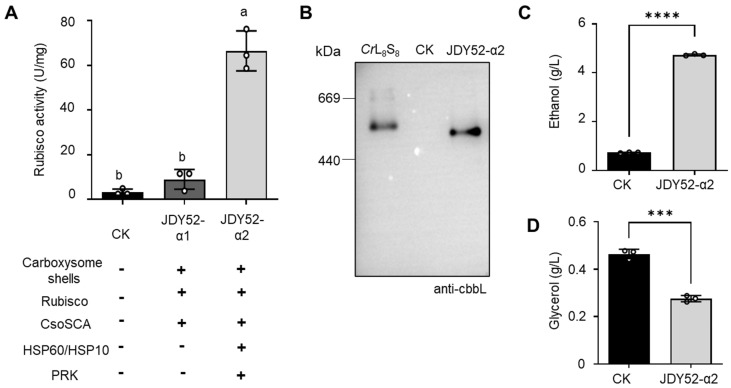
Analyses of Rubisco activity and ethanol/glycerol contents. (**A**) Rubisco enzymatic activity of *S. cerevisiae* strains. Rubisco activity was measured from the cell extracts of different yeast strains by an NADH-based spectrophotometric assay. One unit (1 U) of Rubisco enzyme activity represents the conversion of one nmol NADH per minute per mg protein at 25 °C. CK, JDY52-URR. Tukey’s multiple comparison test was used for statistical analysis. Means denoted by different letters are significantly different from each other (*p* < 0.05). (**B**) Immunoblot detection of Rubisco after native PAGE separation of Rubisco complexes of JDY52-α2. The *Chlamydomonas reinhardtii* Rubisco holoenzyme (*Cr*L8S8) was used for comparison. CK, JDY52-URR. The Rubisco large subunits of both *H. neapolitanus* and *C. reinhardtii* was detected by the anti-CbbL antibody. (**C**,**D**) Contents of ethanol (**C**) and glycerol (**D**) in different strains. The control yeast strain (CK) was grown on SD media, whereas JDY52-α2 was grown on SD lacking Leu and Trp. The contents of ethanol and glycerol were determined by HPLC. In (**C**,**D**), data represented three biological replicates and error bars indicated standard deviations. Student’s *t*-test was used to determine statistical significance (*** *p* < 0.001; **** *p* < 0.0001).

Rubisco carboxylation activity is important for CO_2_ fixation, and its activity was examined from the engineered *S. cerevisiae* strains. In Figure 4A, yeast strain JDY52-α1 demonstrated a level of Rubisco activity similar to the control JDY52-URR. The Rubisco of *H. neapolitanus* belongs to form-I Rubisco (CbbL_8_/CbbS_8_), and the size of Rubisco holoenzyme is about 520 kDa [28]. To facilitate Rubisco folding in *S. cerevisiae*, endogenous *HSP60* and *HSP10* were introduced into strain JDY52-α2. In the presence of carboxysome shells and chaperonin HSP60/HSP10, JDY52-α2 demonstrated significantly increased Rubisco activity compared to JDY52-α1 (Figure 4A). Those data showed that chaperones promoted Rubisco activity. In *S. cerevisiae*, the ability of chaperones to enhance Rubisco activity has been extensively reported. The *E. coli* chaperones GroEL/GroES or *S. cerevisiae* HSP60/HSP10 increased form-II Rubisco (cbbM) activity in a xylose-fermenting *S. cerevisiae* [16,17]. In the JDY52-α2 strain, the activity of Rubisco (CbbL_8_/CbbS_8_) was comparable to the *S. cerevisiae* strains expressing Rubisco (CbbM) and chaperones [16].

The impact of chaperonin HSP60/HSP10 on Rubisco folding was further characterized by native PAGE and immunoblot analysis. In Figure 4B, Rubisco purified from *Chlamydomonas reinhardtii* (the *Cr*L8S8 holoenzyme is about 560 kDa) was used for comparison [29]. *H. neapolitanus* Rubisco holoenzyme (CbbL_8_/CbbS_8_) is around 520 kDa. Molecular weight of *H. neapolitanus* Rubisco expressed in strain JDY52-α2 seemed smaller than *C. reinhardtii* Rubisco holoenzyme (Figure 4B). The data showed that the large (CbbL) and small subunits (CbbS) are able to form the Rubisco holoenzyme (CbbL_8_/CbbS_8_) in the presence of chaperonin HSP60 and HSP10 in *S. cerevisiae*.

The introduced *H. neapolitanus* Rubisco in JDY52-α2 were successfully assembled and demonstrated to be the most active one (Figure 4A,B). We next analyzed the metabolic effect on ethanol production in the engineered strains by HPLC. Compared to the control, JDY52-α2 demonstrated a significant increase (~6.4 fold) of ethanol content (Figure 4C). Consistently, the content of the byproduct glycerol was reduced (~41% reduction) significantly (Figure 4D). Therefore, the introduced carboxysome shells and the engineered PRK and Rubisco pathway effectively enhanced ethanol production in yeast cells.

### 2.4. Characterization of Carboxysome-like Structures

The α-carboxysome shell is mostly composed of CsoS1A hexamers. To differentiate whether the carboxysome shell is assembled correctly, the strain JDY52-CsoS1A expressing CsoS1A was generated. Yeast JDY52-α1 on SD-Leu (SD medium lacking leucine) and JDY52-α2 on SD-Leu-Trp (SD medium lacking both leucine and tryptophan) were observed using the immuno-fluorescence approach. The primary antibody against CsoS1A was used to demonstrate the distribution of this shell protein. For JDY52-CsoS1A, the fluorescence was scattered throughout the cytoplasm of yeast cells (Figure S2). However, for both JDY52-α1 and JDY52-α2 expressing carboxysome gene cassette, fluorescent aggregates were observed near the periphery of cytoplasm (Figure S2). The fluorescent aggregates were around 100–200 nm in diameter, implying the formation of carboxysomes.

**Figure 5:**
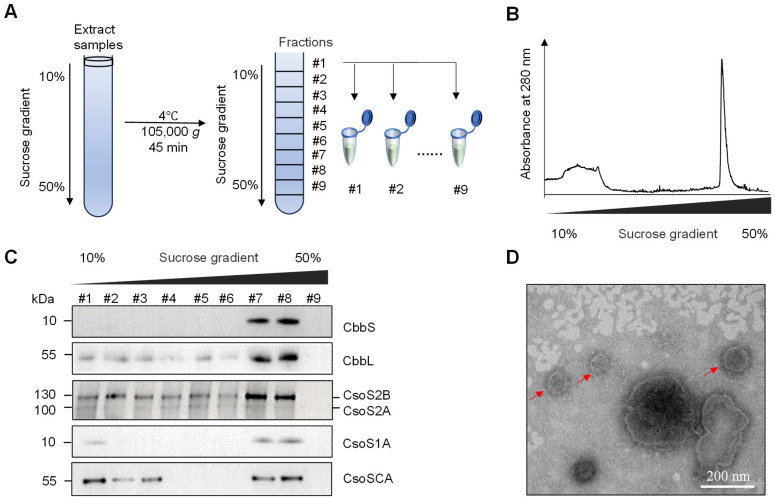
Purification of carboxysomes from the JDY52-α2 strain and EM analyses. (**A**) Diagram of carboxysome purification procedure using sucrose density gradient centrifugation. (**B**) Ultraviolet spectrophotometric measurement of samples after sucrose density centrifugation. Relative absorbance at 280 nm was shown. (**C**) Immunoblot analyses of individual fractions after sucrose density centrifugation. Antibodies against *H. neapolitanus* CbbS, CbbL, CsoS2, CsoS1A and CsoSCA were used. (**D**) Negative-staining transmission electron microscopy analysis of isolated carboxysomes. Scale bar, 200 nm.

To further characterize the α-carboxysome-like structures, carboxysomes were isolated from *S. cerevisiae* strain JDY52-α2 by sucrose density gradient centrifugation following modified procedures from [30,31], as briefly illustrated in Figure 5A. *H. neapolitanus* α-carboxysomes were found to be enriched in fractions 7 and 8 after ultracentrifugation. These two fractions demonstrated higher absorbance at 280 nm (Figure 5B). The carboxysome shell protein (CsoS1A), scaffolding proteins (CsoS2A and CsoS2B) and proteins inside shells (CbbL, CbbS, CsoSCA) were all detectable in fractions 7 and 8 by immunoblotting with corresponding antibodies (Figure 5C), suggesting that carboxysome shells were assembled and that proteins CbbL, CbbS, and CsoSCA were successfully recruited into the shells in *S. cerevisiae*. Fractions 7 and 8 were further analyzed by negative-staining electron microscopy (EM). The reconstructed polyhedral shells with a diameter of 100–200 nm were observed (Figure 5D). Those results showed that carboxysome proteins (CsoS2A, CsoS2B, and CsoS1A) are sufficient for shell assembly in *S. cerevisiae*.

## 3. Discussion

In this study, to reconstruct α-carboxysome in *S. cerevisiae*, *H. neapolitanus* CbbL, CbbS, and CsoSCA proteins were expressed together with α-carboxysome proteins (CsoS1A, CsoS2A, and CsoS2B). In yeast, Ribulose-5-phosphate (R5P) is one of the metabolites of the pentose phosphate pathway [32]. Therefore, the yeast metabolic pathway was engineered by introducing PRK which converts R5P into RuBP. Inside the shell, CsoSCA converts HCO_3_^−^ to CO_2_ and Rubisco efficiently catalyzes carboxylation of RuBP under the low-oxygen environment.

In the strain JDY52-α2, the activity of Rubisco in the presence of chaperonin HSP60/HSP10 was enhanced significantly compared to that of the strain JDY52-α1 without chaperonin (Figure 4A), suggesting that chaperonin HSP60/HSP10 is necessary for Rubisco activity. As demonstrated by native-PAGE analysis in Figure 4B, chaperonin HSP60/HSP10 assisted Rubisco folding in JDY52-α2. Compared to the *C. reinhardtii* Rubisco holoenzyme, it is possible that Rubisco in JDY52-α2 is a holoenzyme, but this conclusion requires further analysis. In the synthesized strain JDY52-α2, PRK catalyzes R5P to RuBP which is the substrate for Rubisco carboxylation reaction. With a low oxygen concentration inside carboxysome structures, CO_2_ is assimilated by Rubisco efficiently, resulting in increased bioethanol production (Figure 4C).

The native α-carboxysomes in *H. neapolitanus* are near 125 nm in diameter [30]. The *H. neapolitanus* α-carboxysome *cso* operon was heterologously expressed and α-carboxysome shells were assembled in different organisms. *Corynebacterium glutamicum* expressing *H. neapolitanus cso* operon or shell proteins formed carboxysome-like structures which were less than 100 nm in diameter [11]. In *E. coli,* the intact α-carboxysome shells of 82–148 nm in diameter were successfully synthesized by expressing relevant shell proteins (CsoS1A/B/C/D, CsoS2, CsoS4A/B) [8]. CsoS2 is necessary for formation of empty carboxysome shells and is also important for the formation of shells encapsulating cargo proteins such as CsoSCA, GFP or hydrogenase [8]. Moreover, in *E. coli*, α-carboxysome proteins (CsoS1A/B/C/D, CsoS2, CsoS4A/B), CsoSCA, Rubisco, and Rubisco activase components (CbbQ/CbbO) assembled into icosahedral shells of 110–140 nm in diameter, and CsoSCA, Rubisco, CbbQ and CbbO were recruited into the recombinant carboxysome shells [33]. Those reports showed that the size of the reconstructed carboxysomes produced in *E. coli* was not affected by the recruited cargo proteins and it was comparable with the native α-carboxysome in *H. neapolitanus*. In this study, only two carboxysome proteins CsoS2 and CsoS1A were expressed in the yeast. As shown in Figure S2 and Table S2, the engineered strain JDY52-α1 and JDY52-α2 contained structures of 100–200 nm, which is similar to that of natural α-carboxysomes in *H. neapolitanus* and the carboxysome shells heterologously expressed in *E. coli* or chloroplasts of *Nicotiana tabacum*. To reconstitute heterologous carboxysomes in *S. cerevisiae* of the comparable size to the natural ones, expressing more shell proteins could be considered in future studies.

The purified carboxysome-like structures demonstrated the existence of carboxysome proteins (CsoS1A, CsoS2A, CsoS2B) and the incorporated proteins (CbbL, CbbS, CsoSCA) (Figure 5C). The immunofluorescence observations in Figure S2, immunoblotting in Figure 5C and the EM observations in Figure 5D together proved that the carboxysome structures were reconstructed in *S. cerevisiae* cells by expressing *csoS1A* and *csoS2* of *H. neapolitanus cso* operon. The carboxysome functions as an autonomous metabolic compartment that achieves modularization and channeling of the carbon fixation reaction. By engineering this module into *S. cerevisiae*, its core function is to create a physically segregated microenvironment within the cytoplasm. This not only enriches the substrate CO_2_ but also isolates the key carbon fixation process from the host’s complex and potentially competing metabolic network, thereby optimizing carbon flux toward the target product, ethanol.

In summary, this study attempted to develop *S. cerevisiae* strains by introducing partial CBB pathway and reconstructing α-carboxysome structures which can provide a low-oxygen environment for CO_2_ fixation. This is the first report of assembling heterologous α-carboxysome in *S. cerevisiae* as far as we know. The engineered strain showed apparent Rubisco activity and significant increase in ethanol content. The ethanol content of the engineered strain JDY52-α2 (4.73 g/L) was approximately six-fold higher than that of WT (0.74 g/L). For the ethanol conversion efficiency, JDY52-α2 (0.24 g/g) was six-fold higher than that of WT (0.04 g/g). For productivity, JDY52-α2 (0.05 g/L/h) was five-fold higher than that of WT (0.01 g/L/h). We anticipate that the engineered strain will serve as a valuable chassis for additional reprogramming of the metabolic pathways to further increase production of ethanol and other metabolites. The novel yeast strain’s capability to utilize carbon dioxide reduces its dependency on sugar during the production process. This advancement not only significantly reduces raw material costs for the fermentation industry but also paves a new green production pathway based on carbon resource recycling, yielding substantial long-term economic and environmental benefits.

## 4. Materials and Methods

### 4.1. Construction of Plasmids and Yeast Strains

The *S. cerevisiae* JDY52-URR [19] was used as a background strain in this study. In JDY52-URR strain, two unique recombination regions (URR1 and URR2) and a selectable marker (His3) were integrated into the JDY52 (MATa his3Δ200 leu2Δ0 lys2Δ0 trp1Δ63 ura3Δ0 met15Δ0) genome at the HO locus on chromosome IV. The coding sequences of carboxysome genes csoS1A, csoS2, cbbL, cbbS and csoSCA of *H. neapolitanus* and PRK (Gene ID: LOC110791203) of *S. oleracea* were codon-optimized and synthesized by GenScript (Nanjing, China). The DNA sequences of HSP60 and HSP10 were amplified from a cDNA library of JDY52-URR strain. The promoter and terminator sequences used in this study were amplified from the genomic DNA of JDY52-URR. Primers used for individual parts are listed in Table S3. The standard procedure was used for yeast transformation.

The biological parts for ORF (open reading frame), PRO (promoter) and TER (terminator) were cloned into the vector pSMART-HCKan by Golden Gate Assembly. The transcriptional unit of PRO-ORF-TER was assembled into POT vectors, generating POT2-pPYK1-cbbS-tNAT5, POT4-pTPI1-cbbL-tCPS1, POT6-pRPL3-csoS2-tADH1, POT8-pENO2-csoS1A-tRPL15A, and POT9-pRPL8-csoSCA-tVMA2, respectively. Then different POT vectors were assembled into the carboxysome cassette, which was composed of the transcriptional units of *csoS1A*, *csoS2*, *cbbL*, *cbbS,* and *csoSCA*. The biological part construction, the transcriptional unit assembly procedures, and carboxysome cassette assembly were performed by following the YeastFab assembly method [19,34].

pSCDuet was modified by ligating fragments URR1-LEU2-URR2 to create pSCDuet-URR. The pSCDuet-URR was linearized to ligate with carboxysome cassette, resulting in pSCDuet-5TU. Then the fragment of URR1-carboxysome cassette-LEU2-URR2 was cleaved from this vector and transformed into yeast strain JDY52-URR. The URR1-carboxysome cassette-LEU2-URR2 was integrated into the yeast genome at the URR sites, leading to the generation of yeast strain JDY52-α1. Positive colonies were selected on SD medium lacking Leu.

The transcriptional units of *PRK*, *HSP60,* and *HSP10* genes were assembled into POT2-pTEF1-PRK-tCYC1, POT4-pPFK1-HSP60-tPRC1, and POT5-pPGK1-HSP10-tNAT1. The module of *PRK-HSP60*-*HSP10* was assembled using the YeastFab method and cloned into vector pRS424 to make pRS424-3TU. The construct pRS424-3TU was transformed into strain JDY52-α1, generating strain JDY52-α2. The positive clones were selected on SD medium lacking both Leu and Trp.

### 4.2. Rubisco Enzymatic Activity Assay

*S. cerevisiae* JDY52-URR was precultured on SD medium lacking His. The engineered yeast strain JDY52-α1 was precultured on SD medium lacking Leu. *S. cerevisiae* strain JDY52-α2 was precultured on SD medium lacking Leu and Trp. The cell cultures were grown at 28 °C until reaching the logarithmic phase. Cells were collected at 1500× *g* at 4 °C for 10 min and rinsed twice with the extraction buffer (100 mM phosphate buffer pH 7.5, 15 mM MgCl_2_, 15 mM NaHCO_3_, protease inhibitor cocktail (Sigma-Aldrich)). The cells were then digested by snailase (30 mg snailase/g cell) at 37 °C for 60 min and centrifuged at 9300× *g* at 4 °C for 10 min. The supernatant was evaluated for Rubisco activity by following the procedures of Rubisco-Bisphosphate Carboxylase/Oxygenase Activity Assay Kit (Solarbio, China). For strains JDY52-α1 and JDY52-α2, the supernatants were frozen at −20 °C for 10–15 min and subsequently thawed on ice to disrupt the carboxysome shells [35], thereby releasing Rubisco for enzymatic activity quantification.

### 4.3. Fermentation Conditions and HPLC Analysis

Yeast strains were cultivated aerobically on SD medium (pH 5.8) at 28 °C and rotated at 170 rpm. Cell cultures were grown for 96 h until reaching the plateau phase.

The contents of ethanol and glycerol were analyzed by using a high-performance liquid chromatography (HPLC) system (LC-20AD, Shimadzu, Japan) equipped with a Refractive Index Detector (RID-10A). Separation was performed on an organic acid analysis column (Bio-Rad Aminex HPX-87H, 300 mm × 7.8 mm) maintained at 60 °C, with a detector temperature of 40 °C. The mobile phase consisted of 5 mM sulfuric acid delivered at a flow rate of 0.5 mL/min. The initial flow rate was set to 0.2 mL/min, and after stabilization of the column temperature and pressure, it was gradually increased to 0.5 mL/min in 0.2 mL/min increments. The injection volume was 20 μL.

### 4.4. Fluorescence Microscopy

The procedure was performed as described previously with modifications [36,37]. The engineered yeast strains were precultured on SD lacking His for strain JDY52-URR, or SD lacking Leu for JDY52-α1, or SD lacking Leu and Trp for JDY52-α2. The preculture was transferred to YPD medium and grew under the same conditions until reaching OD_600_ = 0.8. Cells were collected at 1500× *g* at 4 °C for 5 min, fixed by 4% paraformaldehyde for 30 min, rinsed once with PBS buffer (2.67 mM KCl, 1.47 mM KH_2_PO_4_, 138 mM NaCl, 8.1 mM Na_2_HPO_4_, pH7.4), and rinsed twice with PBS buffer containing 2.4 M sorbitol. The cells were digested by 1% snailase in PBS containing 1.2 M sorbitol at 30 °C for 50 min. The protoplasts were collected at 500× *g* for 5 min and washed three times with PBS containing 2.4 M sorbitol. The appropriate volume of protoplast suspension was dropped onto a poly-L-lysine-coated glass slide. After 20 min, the slide was added with PBS buffer containing 0.2% Triton X-100, 0.5% SDS, 1 mg/mL BSA and 2.4 M sorbitol, and incubated for 6 min. The slide was rinsed with PBS containing 2.4 M sorbitol three times and blocked in PBS buffer containing 2.4 M sorbitol and 1 mg/mL BSA for 1 h. After blocking, the slide was incubated with primary antibody (anti-csoS1A) for 1 h, then rinsed with PBS buffer containing 2.4 M sorbitol three times. Next, the slide was incubated with Abberior STAR RED goat anti-rabbit IgG (Abberior Instruments, Germany) for 1 h and rinsed with PBS buffer containing 2.4 M sorbitol three times. The glass slide was observed using Multi SIM X (NanoInsights-Tech, Beijing, China). Photos were captured with excitation wavelength of 630–650 nm, and the emission signal was detected at 750–800 nm.

### 4.5. Purification of α-Carboxysomes from S. cerevisiae

Purification of synthetic α-carboxysome from S. cerevisiae was conducted following previous protocols with modifications [11,30,38]. The procedures of cell culture and cell wall digestion by snailase were described previously in the “Rubisco enzymatic activity assay” part. The following procedure was performed at 4 °C. After the cell wall digestion, the cell debris was removed through centrifugation at 1000× *g* for 5 min. The protoplasts were lysed in the lysis buffer (containing 0.7 M sorbitol in 50 mM Tris-HCl pH7.5 and 0.2 mM EDTA) and then centrifuged at 2000× *g* for 10 min to discard cell debris. The supernatant was centrifuged at 50,000× *g* for 30 min to concentrate synthetic α-carboxysomes. The resulting pellet containing enriched carboxysomes was resuspended in a small volume TEMB buffer (10 mM Tris-HCl, pH 8.0, 1 mM EDTA, 10 mM MgCl_2_, 20 mM NaHCO_3_) and loaded onto a linear 10–50% sucrose density gradient. The ultracentrifugation was performed at 105,000× *g* for 60 min in a swinging bucket rotor (SW41 Ti, Beckman Coulter). The sucrose fractions were collected at 1.3 mL per fraction from the top. Nine total fractions were collected. Then total protein of each fraction was precipitated by trichloroacetic acid (TCA) method and was analyzed by immunoblotting to assess the presence of corresponding carboxysome proteins by specific antibodies. For the fractions 7 and 8, samples were analyzed by negative-staining transmission electron microscope (FEI Tecnai G2).

### 4.6. Statistical Analysis

Bar graphs were generated using GraphPad Prism 8. Student’s *t*-test or Tukey’s multiple comparison test were used where appropriate to determine statistical significances.

## Data Availability

All data and materials used in this study are available from the corresponding author and will be provided upon reasonable request.

## Supplementary Materials

The following supporting information can be downloaded at https://www.mdpi.com/article/10.3390/ijms26199759/s1 [39].
