# Supplementary material for: Engineering CO2-Fixing Carboxysome into Saccharomyces cerevisiae to Improve Ethanol Production
Source: Int J Mol Sci. 2025 Oct 7;26(19):9759. doi: 10.3390/ijms26199759 (PMC12524633; doi:10.3390/ijms26199759)
Supplement: Supplementary file 1 [file ijms-26-09759-s001.zip › Supplemental Figures.pptx]

## Slide 1
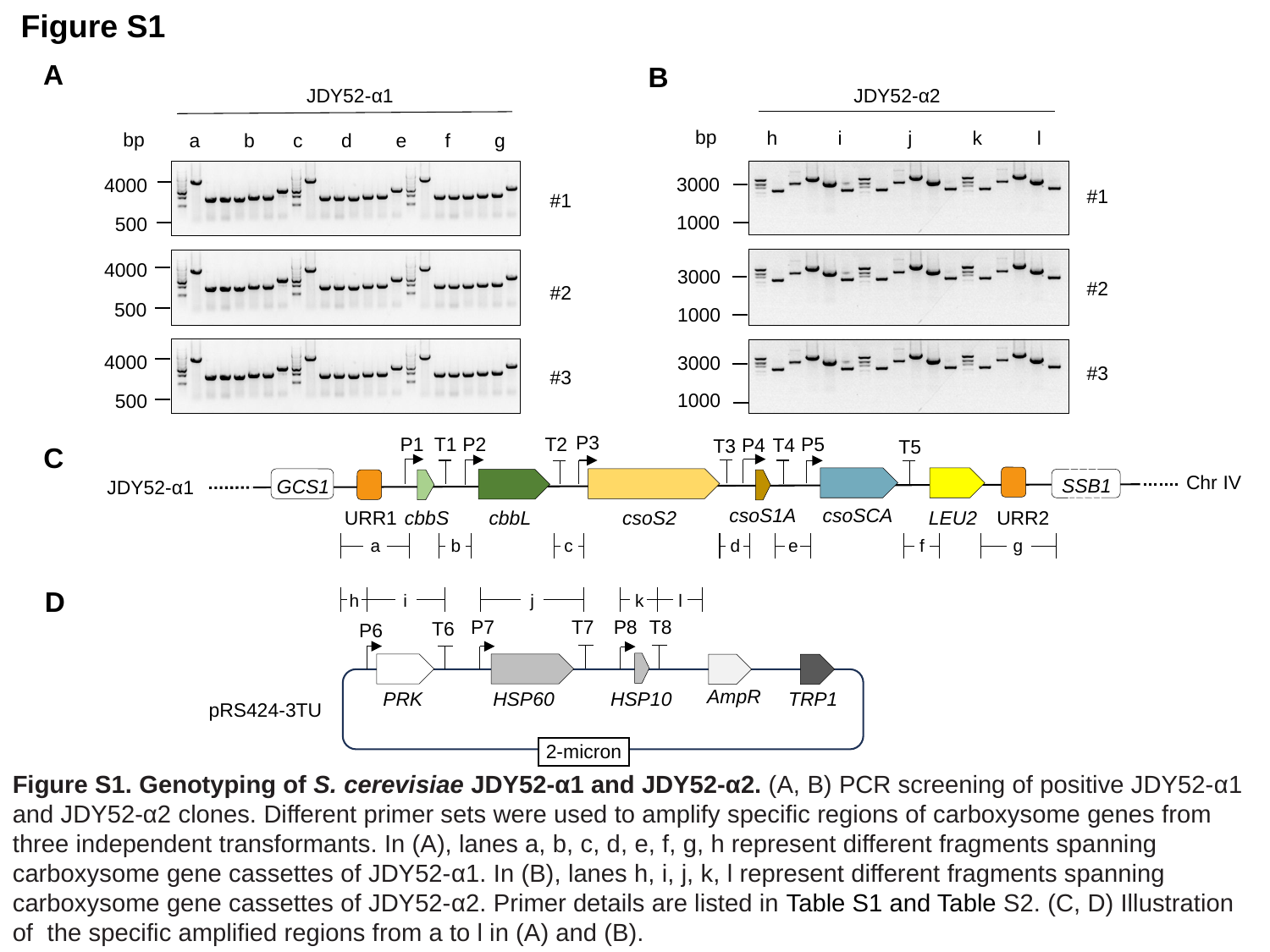

Figure S1
A
B
JDY52-α1
JDY52-α2
 bp
h i j k l
bp
a b c d e f g
 3000
 1000
 4000
500
#1
#1
 4000
500
 3000
 1000
#2
#2
 4000
500
 3000
 1000
#3
#3
Figure S1. Genotyping of S. cerevisiae JDY52-α1 and JDY52-α2. (A, B) PCR screening of positive JDY52-α1 and JDY52-α2 clones. Different primer sets were used to amplify specific regions of carboxysome genes from three independent transformants. In (A), lanes a, b, c, d, e, f, g, h represent different fragments spanning carboxysome gene cassettes of JDY52-α1. In (B), lanes h, i, j, k, l represent different fragments spanning carboxysome gene cassettes of JDY52-α2. Primer details are listed in Table S1 and Table S2. (C, D) Illustration of the specific amplified regions from a to l in (A) and (B).
P3
T1
P2
T2
P5
P1
P4
T4
T3
T5
cbbL
csoS2
cbbS
csoSCA
csoS1A
URR1
LEU2
URR2
C
Chr IV
SSB1
GCS1
JDY52-α1
SSSSbSS1
a
b
c
d
e
f
g
D
h
i
j
k
l
T8
P7
T7
P8
T6
P6
PRK
HSP60
HSP10
AmpR
TRP1
2-micron
222
pRS424-3TU

## Slide 2
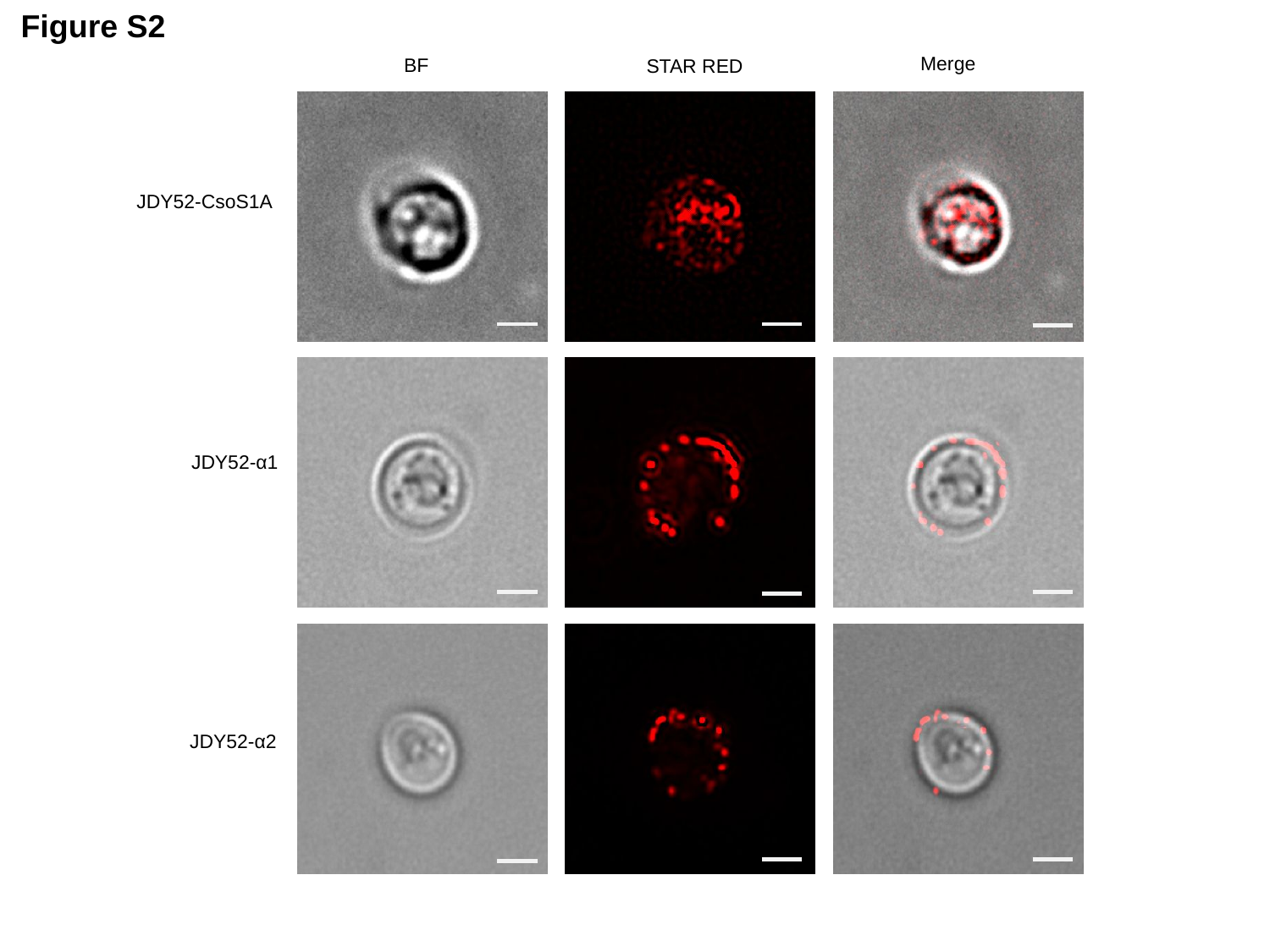

Figure S2
Merge
BF
STAR RED
 JDY52-CsoS1A
JDY52-α1
JDY52-α2

## Slide 3
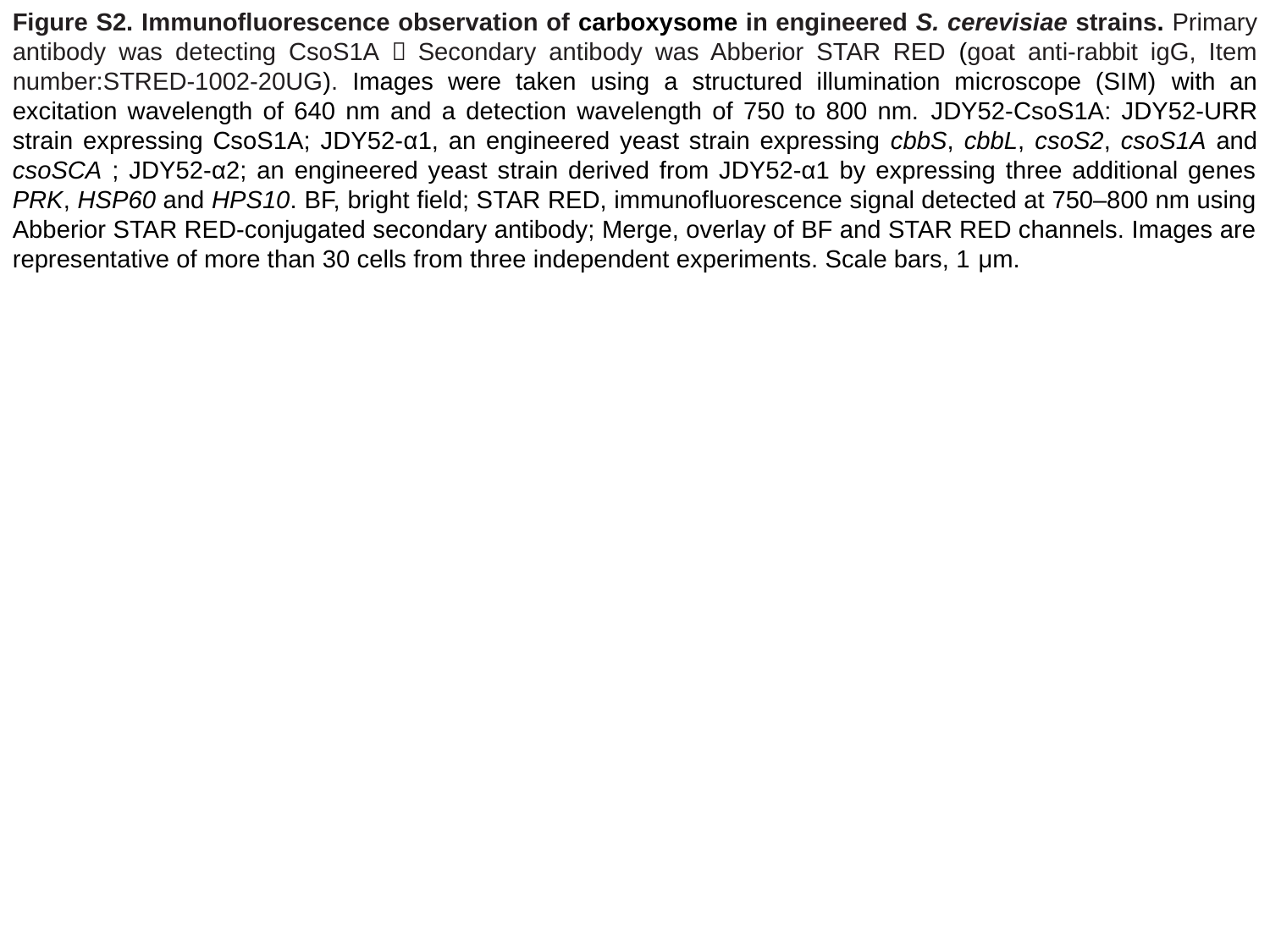

Figure S2. Immunofluorescence observation of carboxysome in engineered S. cerevisiae strains. Primary antibody was detecting CsoS1A；Secondary antibody was Abberior STAR RED (goat anti-rabbit igG, Item number:STRED-1002-20UG). Images were taken using a structured illumination microscope (SIM) with an excitation wavelength of 640 nm and a detection wavelength of 750 to 800 nm. JDY52-CsoS1A: JDY52-URR strain expressing CsoS1A; JDY52-α1, an engineered yeast strain expressing cbbS, cbbL, csoS2, csoS1A and csoSCA ; JDY52-α2; an engineered yeast strain derived from JDY52-α1 by expressing three additional genes PRK, HSP60 and HPS10. BF, bright field; STAR RED, immunofluorescence signal detected at 750–800 nm using Abberior STAR RED-conjugated secondary antibody; Merge, overlay of BF and STAR RED channels. Images are representative of more than 30 cells from three independent experiments. Scale bars, 1 μm.
