# Supplementary material for: Engineering CO2-Fixing Carboxysome into Saccharomyces cerevisiae to Improve Ethanol Production
Source: Int J Mol Sci. 2025 Oct 7;26(19):9759. doi: 10.3390/ijms26199759 (PMC12524633; doi:10.3390/ijms26199759)
Supplement: Supplementary file 1 [file ijms-26-09759-s001.zip › Table S1.pdf]

Table S1. Primers for genotyping

Oligo Name	Sequence 5'->3'	Fragment amplification
HO1-F	CAACGTAAAATTGTGCCTTTGGAC	Fragment a
pPYK1-R	GTGCTGGGTCTCACATCTGTGATGATGTTTTATTTGTT	
NAT5t-F	AGCGTGGGTCTCTTAGCCATTTCTTAACAGATGGCTG	Fragment b
pTPI1-R	GTGCTGGGTCTCACATCTTTTAGTTTATGTATGTGTTTTTTGT	
CPS1t-F	AGCGTGGGTCTCTTAGCGCGCAATGATTGAATAGTC	Fragment c
pRPL3-R	GTGCTGGGTCTCGGAGGATTGTAGCAAAGATTGTAAGGAA	
ADH1term-F	AGCGTGGGTCTCTTAGCCGAATTTCTTATGATTTATGA	Fragment d
pENO2-R	GTGCTGGGTCTCACATCTATTATTGTATGTTATAGTATTAGTTGC	
RPL15At-F	AGCGTGGGTCTCGGGCTGTATTCAAGATATTCTTATCAAAGC	Fragment e
pRPL8B-R	GTGCTGGGTCTCACATCCTTTTCTTTCAGTTATCGTGTTC	
VMA2t-F	AGCGTGGGTCTCTTAGCGAGGACGGTTGCTGAAGAA	Fragment f
LEU2-R	GAACCTTAATGGCTTCGGCTGTG	
LEU2-F	CGCCACTATCTTGTCTGCTGC	Fragment g
SSB1-F	GTCACCAAGGCCATGTCTTCTC	
M13Forward	TGTAAAACGACGGCCAGT	Fragment h
pTEF1-R	GTGCTGGGTCTCACATCTTTGTAATTAAAACTTAGATTAG	
pTEF1-F	AGCGTGGGTCTCGGGCTCAGAAAGCGACCACCCAAC	Fragment i
CYC1term-R	GTGCTGGGTCTCGGAGGGCAAATTAAAGCCTTCGAGC	
pPFK1-F	AGCGTGGGTCTCGGGCTTTTGTTCTCAACAGGCCGT	Fragment j
PRC1t-R	GTGCTGGGTCTCGGAGGGCAGCGATCAGCAATAATGA	

pPGK1-F	AGCGTGGGTCTCGGGCTCTTAATACTAGGATCAGGCA	Fragment k
NAT1t-BsaI-R	GTGCTGGGTCTCgGAGGATATGTGGGATGGTCATCC	
NAT1t-BsaI-F	AGCGTGGGTCTCtTAGCCCTGCAACTCCTCAATGTG	Fragment l
LEU2-R	GAACCTTAATGGCTTCGGCTGTG	
