# Supplementary material for: Engineering CO2-Fixing Carboxysome into Saccharomyces cerevisiae to Improve Ethanol Production
Source: Int J Mol Sci. 2025 Oct 7;26(19):9759. doi: 10.3390/ijms26199759 (PMC12524633; doi:10.3390/ijms26199759)
Supplement: Supplementary file 1 [file ijms-26-09759-s001.zip › Table S2.pdf]

Table S2. Comparison of carboxysomes and Rubisco activity from different organisms

**Organisms**	**Carboxysomes**	**Carboxysome size**	**Functional assay**	**References**
N. tabacum	recombinant α-carboxysome	154.1 ± 36.9 nm	catalytic rate constant for carboxylation*: 3.7 ± 0.1 μmol·mg^{-1}·min^{-1}	Chen *et al*., 2023
H. neapolitanus	α-carboxysome	~130 nm	catalytic rate constant for carboxylation*: 3.6 ± 0.1 μmol·mg^{-1}·min^{-1}	Chen *et al*., 2023; Sun *et al*., 2022
E. coli	recombinant α-carboxysome	120~140 nm	catalytic rate constant for carboxylation*: 4.2 ± 0.2 μmol·mg^{-1}·min^{-1}	Chen *et al*., 2022; Chen *et al*., 2023
E. coli	recombinant α-carboxysome	~100 nm	**	Li *et al*., 2020
S. cerevisiae	recombinant α-carboxysome	100~200 nm	Rubisco activity: 66.53±8.97 U/mg cell extract	This study

* The catalytic rate constant for carboxylation is the maximum carboxylation rate (Vmax) of Rubisco, determined by measuring the initial rates of ^{14}C fixation at varying CO_2 concentrations and fitting the data to the Michaelis-Menten kinetic model. The unit μmol·mg^{-1}·min^{-1} represents micromoles of CO_2 fixation per milligram of protein per minute.

** Under aerobic conditions, *E. coli* expressing Shell-HydA exhibited an H_2 evolution rate of 882.95 ± 154.71 nmol L^{-1} h^{-1}, which is approximately 4.1-fold higher than that of cells expressing free HydA (217.61 ± 25.58 nmol L^{-1} h^{-1}).

References

Chen, T.; Fang, Y.; Jiang, Q.; Dykes, G. F.; Lin, Y.; Price, G. D.; Long, B. M.; Liu, L. N. Incorporation of functional Rubisco activases into engineered carboxysomes to enhance carbon fixation. *ACS Synth. Biol.* **2022**, *11* (1), 154−161.

Chen, T.; Hojka, M.; Davey, P.; Sun, Y.; Dykes, G. F.; Zhou, F.; Lawson, T.; Nixon, P. J.; Lin, Y.; Liu, L. N. Engineering α-carboxysomes into plant chloroplasts to support autotrophic photosynthesis. *Nat. Commun.* **2023**, *14*(1), 2118.

Li, T.; Jiang, Q.; Huang, J.; Aitchison, C. M.; Huang, F.; Yang, M.; Dykes, G. F.; He, H. L.; Wang, Q.; Sprick, R. S.; Cooper, A. I.; Liu, L. N. Reprogramming bacterial protein organelles as a nanoreactor for hydrogen production. *Nat. Commun.* **2020**, *11* (1), 5448.

Sun, Y.; Harman, V. M.; Johnson, J. R.; Brownridge, P. J.; Chen, T.; Dykes, G. F.; Lin, Y.; Beynon, R. J.; Liu, L. N. Decoding the absolute stoichiometric composition and structural plasticity of α-carboxysomes. *mBio* **2022**, *13* (2), e0362921.
