# Supplementary material for: Engineering CO2-Fixing Carboxysome into Saccharomyces cerevisiae to Improve Ethanol Production
Source: Int J Mol Sci. 2025 Oct 7;26(19):9759. doi: 10.3390/ijms26199759 (PMC12524633; doi:10.3390/ijms26199759)
Supplement: Supplementary file 1 [file ijms-26-09759-s001.zip › Table S3.pdf]

Table S3. Primers for plasmid construction		
Oligo Name	Sequence 5'->3'	Purpose
pPYK1-F	AGCGTGGGTCTCGGGCTAATGCTAGTATTTTGGAGAT	Construction and PCR detection of JDY52-α1 strain
pPYK1-R	GTGCTGGGTCTCACATCTGTGATGATGTTTTATTTGTT	
NAT5t-F	AGCGTGGGTCTCTTAGCCATTTCTTAACAGATGGCTG	
NAT5t-R	GTGCTGGGTCTCGGAGGAGTTCGTGATTCTCCTGCA	
pTPI1-F	AGCGTGGGTCTCGGGCTGACCTAACTACATAGTGTTT	
pTPI1-R	GTGCTGGGTCTCACATCTTTTAGTTTATGTATGTGTTTTTTGT	
CPS1t-F	AGCGTGGGTCTCTTAGCGCGCAATGATTGAATAGTC	
CPS1t-R	GTGCTGGGTCTCGGAGGGATTTGACACTTGATTTGACA	
pRPL3-F	AGCGTGGGTCTCTTAGCGAAGTTTTGTTAGAAAATAAATCA	
pRPL3-R	GTGCTGGGTCTCGGAGGATTGTAGCAAAGATTGTAAGGAA	
ADH1term-F	AGCGTGGGTCTCTTAGCCGAATTTCTTATGATTTATGA	
ADH1term-R	GTGCTGGGTCTCGGAGGCCGGTAGAGGTGTGGTCAATAAG	
pENO2-F	AGCGTGGGTCTCGGGCTCGGAAGTGTCTCATAAACT	
pENO2-R	GTGCTGGGTCTCACATCTATTATTGTATGTTATAGTATTAGTTGC	
RPL15At-F	AGCGTGGGTCTCGGGCTGTATTCAAGATATTCTTATCAAAGC	
RPL15At-R	GTGCTGGGTCTCACATCTGCTGATTTATTTGTTTGATCG	
pRPL8B-F	AGCGTGGGTCTCGGGCTGGTGTTCTTCGACTATCAAC	
pRPL8B-R	GTGCTGGGTCTCACATCCTTTTCTTTCAGTTATCGTGTTC	
VMA2t-F	AGCGTGGGTCTCTTAGCGAGGACGGTTGCTGAAGAA	

VMA2t-R	GTGCTGGGTCTCgGAGGAGTTGGACGGCATTCCTCA	
HC-Kan-F	GATCCTTTGATTTTCTACCG	
HC-Kan-R	CTCGATAACTCAAAAAATACG	
M13Forward	TGTAAAACGACGGCCAGT	
M13Reverse	CAGGAAACAGCTATGAC	
Amp-mut-F	TACCGCGTGACCCACGCTC	
Amp-mut-R	GAGCGTGGGTCACGCGGTA	
Amp-F	CTTACTCTAGCTTCCCGG	
Amp-R	TGGCCCCAGTGCTGCAATGATACCGCGTGACCCACGCTCACCGGCT	
pSC-F	GAAGAGATAAATTGCACTGAAATCTAGAGG	
T7-Terminator	GCTAGTTATTGCTCAGCGG	
URR1-BamHI-F	CAGCCAGGATCCGTCATCTAAGCACAGTCGCGCGT	
URR2-AscI-R	CCTGCAGGCGCGCCGATTAGCGTGGCGAGC	
URR2-F	TGCGCTGATCGGAAACGAAT	
pBR322ori-F	GGGAAACGCCTGGTATCTTT	
HO1-F	CAACGTAAAATTGTGCCTTTGGAC	
LEU2-R	GAACCTTAATGGCTTCGGCTGTG	
LEU2-F	CGCCACTATCTTGTCTGCTGC	
SSB1-F	GTCACCAAGGCCATGTCTTCTC	
pTEF1-F	AGCGTGGGTCTCGGGCTCAGAAAGCGACCACCCAAC	Construction and PCR detection of JDY52-α2
pTEF1-R	GTGCTGGGTCTCACATCTTTGTAATTAAAACTTAGATTAG	

pTEF1-mutation-F	ACAGACCGCCTCGTTTCTTTTTC	strain
pTEF1-mutation-R	AAGAAACGAGGCGGTCTGTTTTTTCTTTTCCAAACC	
CYC1-term-F	AGCGTGGGTCTCTTAGCTCATGTAATTAGTTATGTCAC	
CYC1-term-R	GTGCTGGGTCTCGGAGGGCAAATTAAAGCCTTCGAGC	
pPFK1-F	AGCGTGGGTCTCGGGCTTTTGTTCTCAACAGGCCGT	
pPFK1-R	GTGCTGGGTCTCACATCCTTTGATATGATTTTGTTTCAG	
HSP60-BsaI-F	AGCGTGGGTCTCAGATGAAAGAATTGAAATTCGGTGTAG	
HSP60-BsmBI-R	GTGCTGCGTCTCGGCTACATCATACCTGGCATTCCTGGC	
PRC1t-F	AGCGTGGGTCTCTTAGCTAAAGCGTGTATGTGTAGGCA	
PRC1t-R	GTGCTGGGTCTCGGAGGGCAGCGATCAGCAATAATGA	
pPGK1-F	AGCGTGGGTCTCGGGCTCTTAATACTAGGATCAGGCA	
pPGK1-R	GTGCTGGGTCTCACATCTGTTTTATATTTGTTGTAAAAAG	
HSP10-BsaI-F	AGCGTGGGTCTCAGATGTCTATCGTTCCATTGATGGACCG	
HSP10-BsmBI-R	GTGCTGCGTCTCGGCTAGTCCTTGGCAATCTTAGCC	
NAT1t-BsaI-R	GTGCTGGGTCTCGGAGGATATGTGGGATGGTCATCC	
NAT1t-BsaI-F	AGCGTGGGTCTCTTAGCCCTGCAACTCCTCAATGTG	
